# Moving towards universal health coverage: advanced practice nurse competencies

**DOI:** 10.1590/1518-8345.2901.3132

**Published:** 2019-07-18

**Authors:** Judy Honig, Susan Doyle-Lindrud, Jennifer Dohrn

**Affiliations:** 1 Columbia University, School of Nursing, New York, NY, EUA

**Keywords:** Advanced Practice Nursing, Curriculum, Competencies, Primary Healthcare, Education, Consensus, Prática Avançada de Enfermagem, Currículo, Competência, Atenção Primária à Saúde, Educação, Consenso, Enfermería de Práctica Avanzada, Currículo, Competencia, Atención Primaria de Salud, Educación, Consenso

## Abstract

**Objective:**

this paper aims to describe the first phase of a project whose general goal was to develop a consensus-based set of advanced practice nurse competencies applicable to Latin American countries and, based on these competencies, produce an advanced practice nurse curricular prototype adapted to Latin American countries. The project was framed in a competency-based approach to advanced practice nursing education. The specific aims of the first phase of the project described in this paper were: 1) to identify a set of potential advanced practice nurse competencies that would serve as the template for Core Advanced Practice Nurse Competencies in Latin American countries and 2) to establish consensus for Core Advanced Practice Nurse Competencies in Latin American countries.

**Method:**

advanced practice nurse competencies were derived from a comprehensive review of published competencies and informed the development of a survey designed to assess the relevance of advanced practice nurse competencies in Latin American countries. The survey was distributed to nurse leaders and nurse educators. Data were analyzed using descriptive statistics.

**Results:**

consensus for Core Competencies was established.

**Conclusion:**

the Core Advanced Practice Nurse Competencies presented can provide a structured framework to build educational programs aligned to the needs of the regional environment.

## Abstract (pt)

**Objetivo:**

o objetivo deste artigo é descrever a primeira fase de um projeto que teve como finalidade geral desenvolver um conjunto de competências de práticas avançadas de enfermagem baseadas em consenso aplicáveis em países da América Latina e, usando tais competências, produzir um protótipo curricular de práticas avançadas de enfermagem a serem adaptadas em países da América Latina. O projeto foi enquadrado em uma abordagem baseada em competências para o ensino de práticas avançadas de enfermagem. Os objetivos específicos da primeira fase do projeto descritos neste artigo foram: 1) identificar um conjunto de competências em enfermagem para práticas avançadas que serviriam como modelo para as Competências Essenciais de Enfermeiros de Práticas Avançadas em países da América Latina e 2) estabelecer um consenso sobre Competências Essenciais de Enfermeiros de Práticas Avançadas em países da América Latina.

**Método:**

as competências de enfermeiros de práticas avançadas foram derivadas de uma revisão abrangente das competências publicadas e informaram o desenvolvimento de uma pesquisa projetada para avaliar a relevância das competências de enfermeiros de práticas avançadas nos países da América Latina. A pesquisa foi distribuída entre enfermeiros líderes e enfermeiros educadores. Os dados foram analisados por meio de estatística descritiva.

**Resultados:**

foi estabelecido um consenso sobre Competências Essenciais.

**Conclusão:**

apresentam-se as Competências Essenciais de Enfermeiros de Práticas Avançadas que podem fornecer um quadro estruturado para construir programas educacionais alinhados com as necessidades do ambiente regional.

## Abstract (es)

**Objetivo:**

el objetivo de este documento es describir la primera fase de un proyecto, cuyo objetivo general es desarrollar un conjunto basado en consenso de competencias de práctica avanzada de enfermería aplicables en los países de América Latina y utilizando las competencias, producir una enfermera con práctica avanzada de prototipo curricular para la adaptación en países latinoamericanos. El proyecto se encuadró en un enfoque basado en competencias para la educación de enfermería de práctica avanzada. Los objetivos específicos de la primera fase del proyecto descritos en este documento fueron: 1) identificar un conjunto de competencias potenciales de enfermería de práctica avanzada que servirían como un modelo para las Competencias Básicas de Enfermería de Práctica Avanzada en países latinoamericanos y 2) establecer consenso para las Competencias Básicas de Enfermería de Práctica Avanzada en países latinoamericanos.

**Método:**

las competencias de práctica avanzada de enfermería se derivaron de una revisión exhaustiva de las competencias publicadas e informaron el desarrollo de una encuesta diseñada para evaluar la relevancia de las competencias de práctica avanzada de la enfermera en los países de América Latina. La encuesta fue distribuida a enfermeras líderes y enfermeras educadoras. Los datos fueron analizados utilizando estadística descriptiva.

**Resultados:**

se estableció el consenso para las Competencias Básicas.

**Conclusión:**

se presentan las Competencias Básicas de Enfermería de Práctica Avanzada que pueden proporcionar un marco estructurado para construir programas educativos alineados con las necesidades del entorno regional.

## Introduction

An expanded role in nursing is emerging globally, responding to the need for increased human resources in support of a unanimous United Nations resolution to move forward the goal of Universal Health Coverage (UHC) and Primary Health Care (PHC). Universal Health Coverage was adopted by the World Health Organization (WHO) and a global coalition of over 500 health organizations. The resolution states that UHC provides access to an efficient and affordable health system staffed with well-trained health workers^(^ ^1^ ^)^. In turn, according to the Declaration of Alma-Ata (1978), Primary Health Care “is essential health care based on practical, scientifically sound and socially acceptable methods and technology made universally accessible to individuals and families in the community through their full participation and at a cost that the community and the country can afford^(^ ^2^ ^)^.” Together, UHC and PHC provide the overarching objectives for global strategic planning. They serve as a clarion call for countries to strengthen their health systems and establish new models of care, and build health professional capacity in primary health care. Evidence supports that nurses who take on advanced roles are able to provide primary health care to large populations^(^ ^3^ ^)^. One strategy to achieve UHC and PHC is to enrich and maximize the scope of nursing practice with capacity to meet patient-centered primary health care needs.

This important global initiative of UHC and PHC faces persistent challenges. Some of the contributing factors include extreme global poverty, gaps in service delivery, health disparities, emerging and existing vulnerable populations, the growing impact of social determinants of health, changing demography, aging of populations, birth and mortality rates, and the prevalence of chronic illness along the lifespan in addition to the high number of people affected by infectious diseases. A response to these factors necessitates well prepared health professionals, educated and empowered to respond to and improve population health outcomes. Advanced practice nurses are in position to meet these challenges. A basis on advanced nursing competencies provides clarity and structure regarding the role of these professionals so as to build Advanced Practice Nursing (APNs) education programs. The identification of competencies of advanced practice nurses is an important first step.

In 2008, the International Council of Nurses (ICN) published an important document for the international nursing community. The *ICN Scope of Practice, Standards and Competencies of the Advanced Practice Nurse* ^(^ ^4^ ^)^ was proposed as a framework to be used as nations develop APN roles, scopes of practice, and education programs. Standards and competencies are intentionally stated in broad terms so as to allow for refinement and revisions as nations develop these roles and propose APN education. Although APN is growing globally, region-relevant standards of practice and APN competencies are often not well established during the development stages. The lack of clarity results in divergent definitions of APN practice and its core competencies. The advanced practice nurse movement is at various stages of development throughout the world, including Europe, Asia, Africa, North America, New Zealand and Australia, which results in advanced practice nurses taking on different roles, scopes of practice, definitions, and names^(^ ^5^ ^-^ ^6^ ^)^. Some countries have well established APN roles and competencies. For example, in the United States, advanced practice nurses are recognized and highly utilized providers and have a well-defined scope of practice, core competencies, and educational requirements. They provide frontline primary health care, a central need to meet UHC. Central and South America and some of the Caribbean countries are not yet significantly represented in the advanced practice nursing movement. The project described in the present paper was carried out to develop core competencies that will serve as a foundation and template for individual Latin American countries to build an educational and regulatory framework for APN.

In 2013, the Pan American Health Organization/World Health Organization (PAHO/WHO) issued a resolution: *Human Resources for Health: Increasing access to qualified health care workers in primary health care-based health systems* (CD52.R13)^(^ ^7^ ^)^. A major component of the resolution is to build health professional capacity in primary health care and maximize the scope of practice according to competencies. A sound approach includes increased capacity by educating nurses with expanded scopes of practice to lead and take an active role in the movement for UHC and PHC. To contribute to this end, a two-phase project was undertaken. The first phase consists in establishing APN core competencies in Latin America countries (LAC). The second phase aims to use these established competencies as the blueprint to build a prototypical competency-based curriculum for use in LAC. This paper describes the results of the first phase of the project.

This first phase was included in a competency-based approach to nursing education, specifically, APN competencies. The process that was undertaken to establish APN core competencies in LAC is outlined. At first, a comprehensive review of published APN competencies was conducted, a set of APN competencies from the review was derived and used to support a survey designed to delineate advanced practice and competencies in relation to universal health coverage and primary health care in LAC. The APN Competency Survey was distributed among nurse educators and nurse leaders in LAC to determine a set of APN Core Competencies in LAC.

## Method

The aim of the study is to identify a set of potential APN competencies that would serve as the template for APN Core Competencies in LAC. A set of potential APN competencies was derived from a comprehensive review of established and published APN competencies. Using the PubMed database and searching for grey literature, the research team collated documents aimed at identifying APN competencies^(^ ^8^ ^-^ ^13^ ^)^. Using an iterative and deductive process, two experts in competency-based advanced practice education collated the data at the individual competency level. They worked independently for the collation of statements and then collaborated on the categorization of the statements from August 2015-January 2016. The competency statements were clustered, redundancies were eliminated, comparable statements were combined in succinct statements, outliers were eliminated, and themes were described.

The set of derived competencies were conceptualized into four domains: 1) clinical care; 2) inter-professional and patient-centered communication; 3) context of care; and 4) evidence-based practice. This draft of derived competencies served as the foundation for the survey that was created to better understand the relevance of each competency in the context of primary health care in LAC and to establish consensus.

The survey incorporated the derived APN competencies and was designed to delineate advanced practices and competencies in relation to UHC and PHC in LAC. The survey instrument was developed between January 2016- March 2016. The English version was piloted in February 2016, and the feedback from the participants led some items to be clarified and modified. The revised English survey was translated into Spanish using the translation/back translation method. The Spanish version was piloted in March 2016, after which some minor modifications were made. The final versions were entered into a web-based platform for distribution.

The survey instrument was designed by the researchers and consisted of 47 items. The breakdown of sections is as follows: The first item is the consent form, which must be answered in the affirmative in order to proceed, followed by nine multiple choice items including background and demographic information of the respondent and items about nurse and APN capacity. The remaining 37 items are presented on a five-point Likert scale. Seven items addressed assumptions about APN, and 26 items addressed the competency domains, including: clinical care, inter-professional and patient centered communication, context of care, and evidence-based practice. The final four items pertain to essentials of primary health care. In the case of the items on competency domains, the respondents were asked to rate each competency on a scale from “strongly disagree” to “strongly agree” as 1) a valued component of primary health care and universal access, and 2) a realistic goal for advanced practice nursing in their country. The survey was password protected. The Columbia University Medical Center Institutional Review Board IRB approved the study.

The web-based electronic survey was set up so that the respondents can access the survey via a link and all responses remain anonymous and are not asked to provide any identifiable data. The survey was sent to nurse educators and leaders in nursing services to establish consensus about advanced practice competencies in LAC countries. Using the snowball sampling technique, the respondents were asked to forward the survey to their professional networks. The survey was launched in April 2016 for distribution and closed in August 2016.

Descriptive statistics including frequencies and rates was used to summarize the data.

## Results

Eighty-nine nurses from a total of ten LAC, including Argentina, Brazil, Chile, Columbia, Costa Rica, Cuba, Mexico, Nicaragua, Uruguay, and El Salvador, responded to the survey.

More than half of the respondents (N=79) indicated their primary role as nurse faculty member. Of the remaining respondents, 9% were Directors of Nursing, 8% were Chief Nursing Officers, 6% were professional nursing organization officers, and 6% were Deans of schools of nursing.

All of the respondents identified the following nursing education programs in their county: baccalaureate degree and academic and professional masters programs.

The underlying assumptions regarding advanced practice nursing were all reported above four on the five-point Likert scale, with five being the strongest agreement (N=53). The most important components relevant to primary health care included health promotion, disease prevention, diagnosis and management of chronic illnesses, and population health. Of the respondents, 59% ranked infants and children as the highest priority population for primary health care service, followed by 25% who ranked geriatrics as the highest priority. The leading relevant contents were: pregnancy care, health care maintenance, respiratory, cardiovascular, female reproductive, nutritional and digestive, and behavioral/emotional health disorders.

### Advanced Practice Nurse Core Competencies

**Figure 1:**
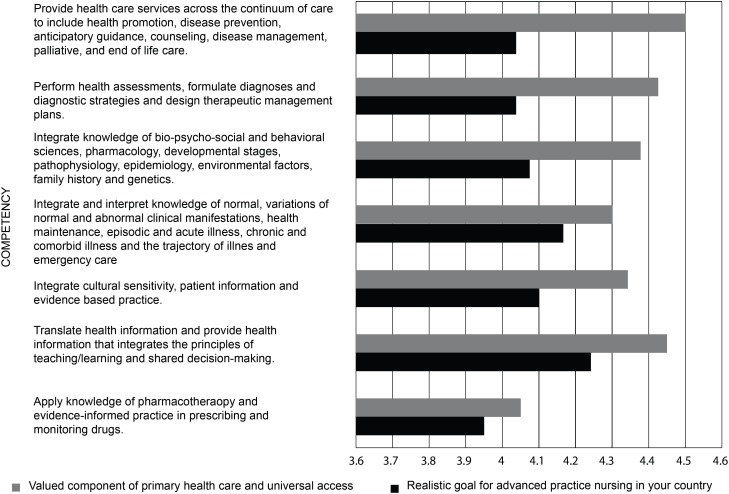
Domain I: Clinical Care (N=60)

**Figure 2:**
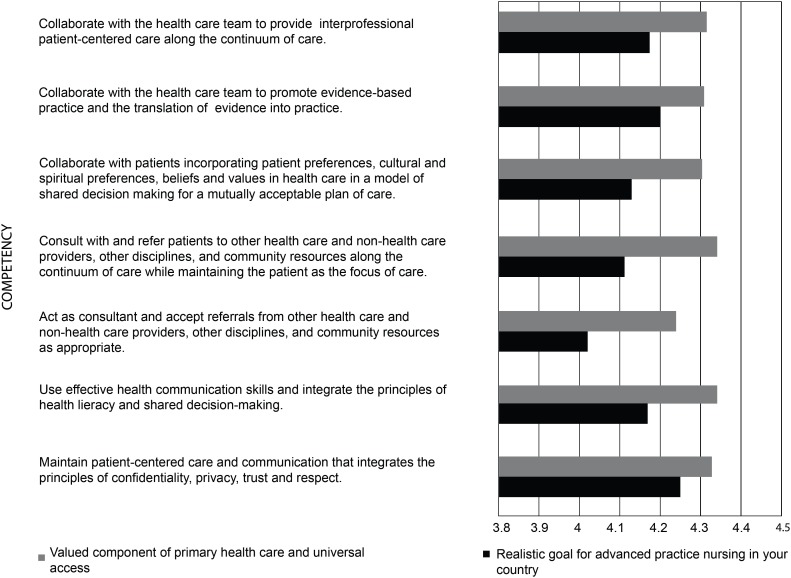
Domain II: Interdisciplinary and Patient-Centered Communication (N=59)

**Figure 3:**
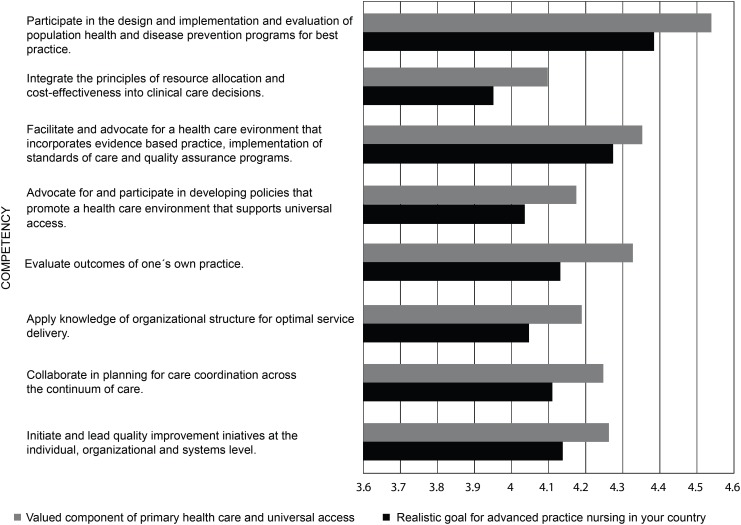
Domain III: Context of Care (N=57)

**Figure 4:**
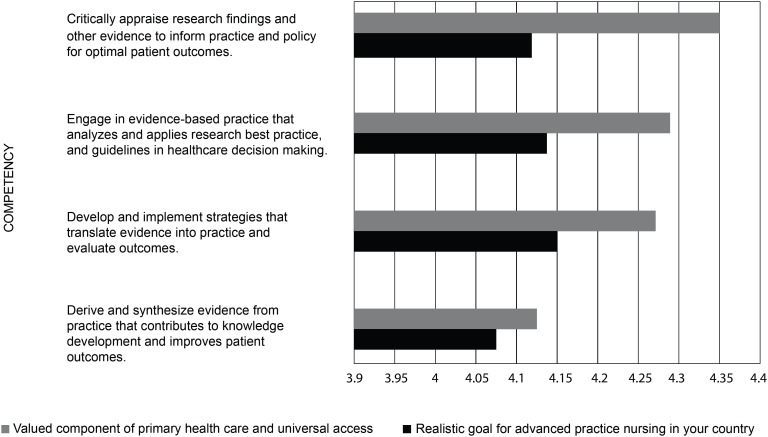
Domain IV: Evidence-Based Practice (N=57)

In the questions related to advanced practice nursing competencies, the respondents rated each competency on a scale from “strongly disagree” to “strongly agree” as 1) a valued component of primary health care and universal access, and 2) a realistic goal for advanced practice nursing in their country. The competencies were rated agree/strongly agree (above four on the five-point Likert scale) in all four domains. Figures 1-4 display the strength of agreement in each domain. The total mean score for each domain as a valued component of primary health care and universal health access ranged between 4.28 and 4.35, and the Domain I (Clinical Care) had the highest mean score. The mean score for each domain as a realistic goal for advanced practice nursing in their country ranged from 4.09-4.15, and the Domain I (Clinical Care) received the lowest score.

## Discussion

The results of this study add to the body of work that seeks to advance the role of nursing and contribute to the goal of UHC and PHC. Consensus regarding the PHC and APN competencies provides a framework to build education programs for advanced practice nurses and provides a structure for regulation. The implementation strategy for advanced practice in LAC includes “adaptation of the existing framework, utilization of recent research, and application of knowledge of experts on advanced practice nursing and primary health care”^(^ ^14^ ^)^. Historically, advanced practice roles evolved to fill gaps as apprenticeship, non-degree models^(^ ^15^ ^)^ and, in some countries, transitioned into formal academic programs. The establishment of formal degree programs relies on the framework of consensus-based APN competencies that drive the curricular content and that can be systematically regulated.

A recent study examined the extent to which nursing education in schools of nursing (N=246) in 25 LAC included primary health care in LAC. The focus of the survey was on the preparation of baccalaureate nursing students to meet the primary health care demands. The American Association of Colleges of Nursing (AACN) Baccalaureate curriculum standards guided the curricula in the schools that participated in the study. The authors reported that most of the students’ clinical hours are attained in hospital settings. They found that the curricula included health care systems, public health, and patient safety. This knowledge is the strong foundation upon which to build a graduate program for advanced practice nurses. However, the identified curricular content that was less emphasized and/or missing included information on technology, environmental health, social justice, advocacy, and care coordination, to name a few. The fact that the programs were guided by the AACN Essentials has implications for this study, because several of the documents addressing competencies that were used to build the APN Core Competencies in the survey can be mapped to the AACN Essentials. In addition, the less emphasized area of content was included in this survey and reflects competencies in complex decision-making, evidence-based practice, and an understanding of the context of care^(^ ^16^ ^)^. Therefore, the APN core competencies included in this study build on the strengths of the existing nursing education and include untapped knowledge and content for primary health care.

Nursing leaders (N=173) were surveyed to better understand the scenario in LAC with respect to advanced practice nurses. The participants identified the lack of regulation for advanced practice nurses or plans to develop such regulation. Ninety percent of the participants (N=155) agreed that advanced practice nurses would be very beneficial to their country and its population. Although they reported that students would be interested in pursuing an advanced degree for the advanced practice role, faculty preparedness to teach in such programs was identified as a concern^(^ ^17^ ^)^. This study confirms that LAC are at the initial stage of APN evolution in terms of regulation and faculty readiness to teach in graduate programs, but highlights that there is a pipeline of interested baccalaureate students throughout LAC and that advanced practice nurses would be an asset to the population and the health care system. Besides providing a framework for advanced practice nursing curricula, a set of APN core competencies would also provide guidelines for APN scope of practice to support the regulatory system.

**Figure 5:**
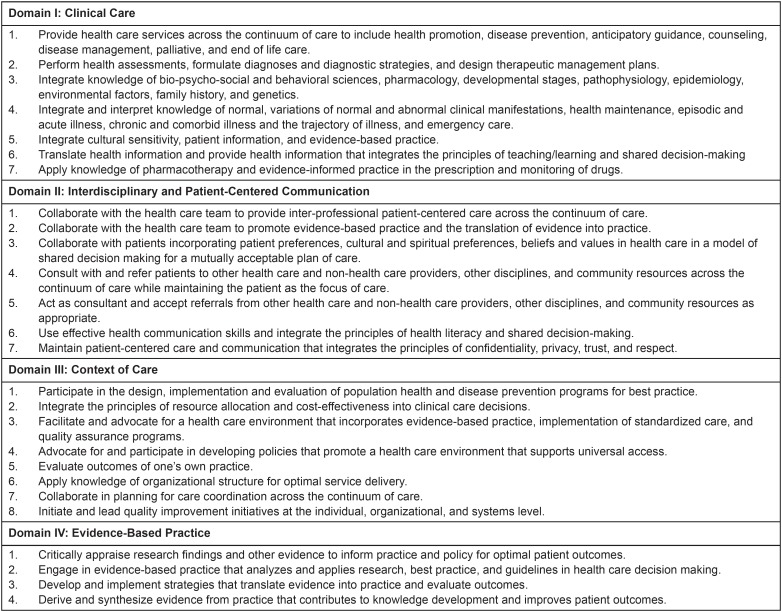
Core Advanced Practice Nurse Competencies for Latin American Countries

With respect to the readiness of LAC to implement the advanced practice nurse role to promote universal health coverage, current research supports the expanded role. Several important facilitating factors are in place, including baccalaureate programs and the potential for master's programs, a pipeline of prospective advanced practice students, and support for the role to promote universal health coverage. The results of this study indicate consensus among the respondents, who are nurse leaders in LAC. The competencies are stated in general rather than prescriptive terms. The competencies are meant to provide a structured framework to build educational programs that are aligned to the regional and/or country-level environment. Further refinement of the Core Advanced Practice Nurse Competencies is needed to tailor the competencies to the country/region. The Core Advanced Practice Nurse Competencies for LAC also form the foundation for future work in developing advanced practice nurse capacity in LAC. Ministries of health, regulatory authorities/boards, and educational institutions can use the competencies as a framework to build context-specific APN competencies and to tailor context-specific curricula. Figure 5 outlines the Core Advanced Practice Nurse Competencies.

## Conclusion

The small sample size with participants from 10 countries represents a limitation of this study, which implies that the results must be interpreted with caution. The number of countries and the heterogeneity among LAC represent a barrier to making statements that apply throughout LAC. More than half of the participants were predominantly nurse faculty members, which may have skewed the results. It is important to recognize that the Core APN Competencies is a starting point to build context-specific competencies from which curricula and regulation can emerge.

The next step for the Core APN Competencies is their specific modification in each country- for the best adequacy within educational programs, faculty resources, and primary health care gaps, as well as optimization of the country's strengths while addressing the challenges. Furthermore, the competencies will be further described using learner-specific performance objectives. Sample content will be included, and this will aid the development of the curriculum. The final document will be disseminated through publications and webinars.

By using the consensus-based APN Core Competencies for LAC, different regions can develop a model curriculum by mapping content to support the competencies. By augmenting the competencies to include content areas to support the curriculum, innovative curricula will emerge that fit the context of each country. Moreover, the regions can further refine the APN Core Competencies according to local relevance in their health systems. One strategy may be to conduct a Delphi study with nurse leaders, public health nurses, and educators in the region. These region-specific competencies can be mapped to the content to build a model curriculum integrated with graduate education. Core APN Competencies provide a framework for LAC to tailor APN education programs, develop a template for a competency-based APN curriculum, and provide an infrastructure for regulatory processes.
